# Amyloid Aggregates Are Localized to the Nonadherent Detached Fraction of Aging Streptococcus mutans Biofilms

**DOI:** 10.1128/spectrum.01661-22

**Published:** 2022-08-11

**Authors:** Elena Yarmola, Ivan P. Ishkov, Nicholas M. di Cologna, Megan Menashe, Robert L. Whitener, Joanna R. Long, Jacqueline Abranches, Stephen J. Hagen, L. Jeannine Brady

**Affiliations:** a Department of Oral Biology, University of Florida, Gainesville, Florida, USA; b Department of Physics, University of Florida, Gainesville, Florida, USA; c Department of Biochemistry and Molecular Biology, University of Florida, Gainesville, Florida, USA; Griffith University

**Keywords:** *Streptococcus mutans*, adhesins, amyloid, biofilms

## Abstract

The number of bacterial species recognized to utilize purposeful amyloid aggregation within biofilms continues to grow. The oral pathogen Streptococcus mutans produces several amyloidogenic proteins, including adhesins P1 (also known as AgI/II, PAc) and WapA, whose truncation products, namely, AgII and AgA, respectively, represent the amyloidogenic moieties. Amyloids demonstrate common biophysical properties, including recognition by Thioflavin T (ThT) and Congo red (CR) dyes that bind to the cross β-sheet quaternary structure of amyloid aggregates. Previously, we observed amyloid formation to occur only after 60 h or more of S. mutans biofilm growth. Here, we extend those findings to investigate where amyloid is detected within 1- and 5-day-old biofilms, including within tightly adherent compared with those in nonadherent fractions. CR birefringence and ThT uptake demonstrated amyloid within nonadherent material removed from 5-day-old cultures but not within 1-day-old or adherent samples. These experiments were done in conjunction with confocal microscopy and immunofluorescence staining with AgII- and AgA-reactive antibodies, including monoclonal reagents shown to discriminate between monomeric protein and amyloid aggregates. These results also localized amyloid primarily to the nonadherent fraction of biofilms. Lastly, we show that the C-terminal region of P1 loses adhesive function following amyloidogenesis and is no longer able to competitively inhibit binding of S. mutans to its physiologic substrate, salivary agglutinin. Taken together, our results provide new evidence that amyloid aggregation negatively impacts the functional activity of a widely studied S. mutans adhesin and are consistent with a model in which amyloidogenesis of adhesive proteins facilitates the detachment of aging biofilms.

**IMPORTANCE** Streptococcus mutans is a keystone pathogen and causative agent of human dental caries, commonly known as tooth decay, the most prevalent infectious disease in the world. Like many pathogens, S. mutans causes disease in biofilms, which for dental decay begins with bacterial attachment to the salivary pellicle coating the tooth surface. Some strains of S. mutans are also associated with bacterial endocarditis. Amyloid aggregation was initially thought to represent only a consequence of protein mal-folding, but now, many microorganisms are known to produce functional amyloids with biofilm environments. In this study, we learned that amyloid formation diminishes the activity of a known S. mutans adhesin and that amyloid is found within the nonadherent fraction of older biofilms. This finding suggests that the transition from adhesin monomer to amyloid facilitates biofilm detachment. Knowing where and when S. mutans produces amyloid will help in developing therapeutic strategies to control tooth decay and other biofilm-related diseases.

## INTRODUCTION

Like human societies in which individuals live in communities that better enable them to contend with their environments, bacteria rely on cooperative activities and acellular architectural components to facilitate community survival under changing conditions (1). Bacteria within biofilms evade host immunity and resist environmental stressors and antibiotic treatment significantly better than their planktonic counterparts (2). In addition to bacterial cells, biofilms contain complex extracellular matrices built with polysaccharides, proteins, nucleic acids, lipids, and other biomolecules, cumulatively called extracellular polymeric substances (EPS), which play key structural and functional roles related to surface adhesion, mechanical stability, and intercellular interactions within the microenvironment (3–7). Proteins present in extracellular matrices include adhesins, subunits of flagella and pili, secreted extracellular proteins, and protein cargo of extracellular membrane vesicles. They participate in cell attachment and migration along surfaces and can contribute to the biofilm’s structural stability (8).

Amyloids are insoluble fibrillar aggregates derived from soluble proteins. Structurally, amyloids are comprised of a stable assembly of β-sheets stacked perpendicular to a fiber axis and share certain biophysical properties (9). Amyloid fibers have a tensile strength comparable to steel and are protease and detergent resistant (10, 11). Their conserved physical and morphological properties suggest that the ability to assemble into fibers is likely a widespread ancient biological process (12). Amyloids were recognized originally in the context of disease and were initially thought to represent exclusively a product of detrimental, self-templated protein misfolding. However, it has more recently been discovered that living organisms ranging from bacteria to humans produce functional amyloids that accomplish specific biological tasks (13). It is increasingly recognized that numerous microbial species utilize purposeful amyloid aggregation to create additional structural and functional tools within biofilm environments. Specifically, bacterial amyloids have been reported to contribute to functions of adhesion, biofilm development, genetic competence, quorum sensing and cell density regulation, antimicrobial activity, signaling pathways, host interactions, and aerial hyphae formation of pathogenic, commensal, and environmental microorganisms (13–24). It is likely that this list will continue to grow.

The oral microbial pathogen Streptococcus mutans is a keystone pathogen of dental caries and is a quintessential biofilm dweller (25). Four different S. mutans proteins have been identified that have the capacity to form amyloid. Two of them, namely, P1 (also called AgI/II or PAc) and wall-associated protein A (WapA), are cell wall-localized adhesins that are covalently attached to the peptidoglycan via the transpeptidase Sortase A (26). The naturally occurring truncation products of P1 and WapA, denoted AgII and AgA, respectively, are amyloidogenic, as are the purified full-length adhesins (27, 28). In addition, another Sortase A substrate, the collagen binding protein Cnm, associated with serotype e, *f*, and *k* and a subset of serotype *c* strains, also forms amyloid (29). Lastly, the secreted protein Smu_63c, whose deletion markedly increases biofilm cell density and genetic competence, is also capable of amyloid aggregation (27).

**FIG 1:**
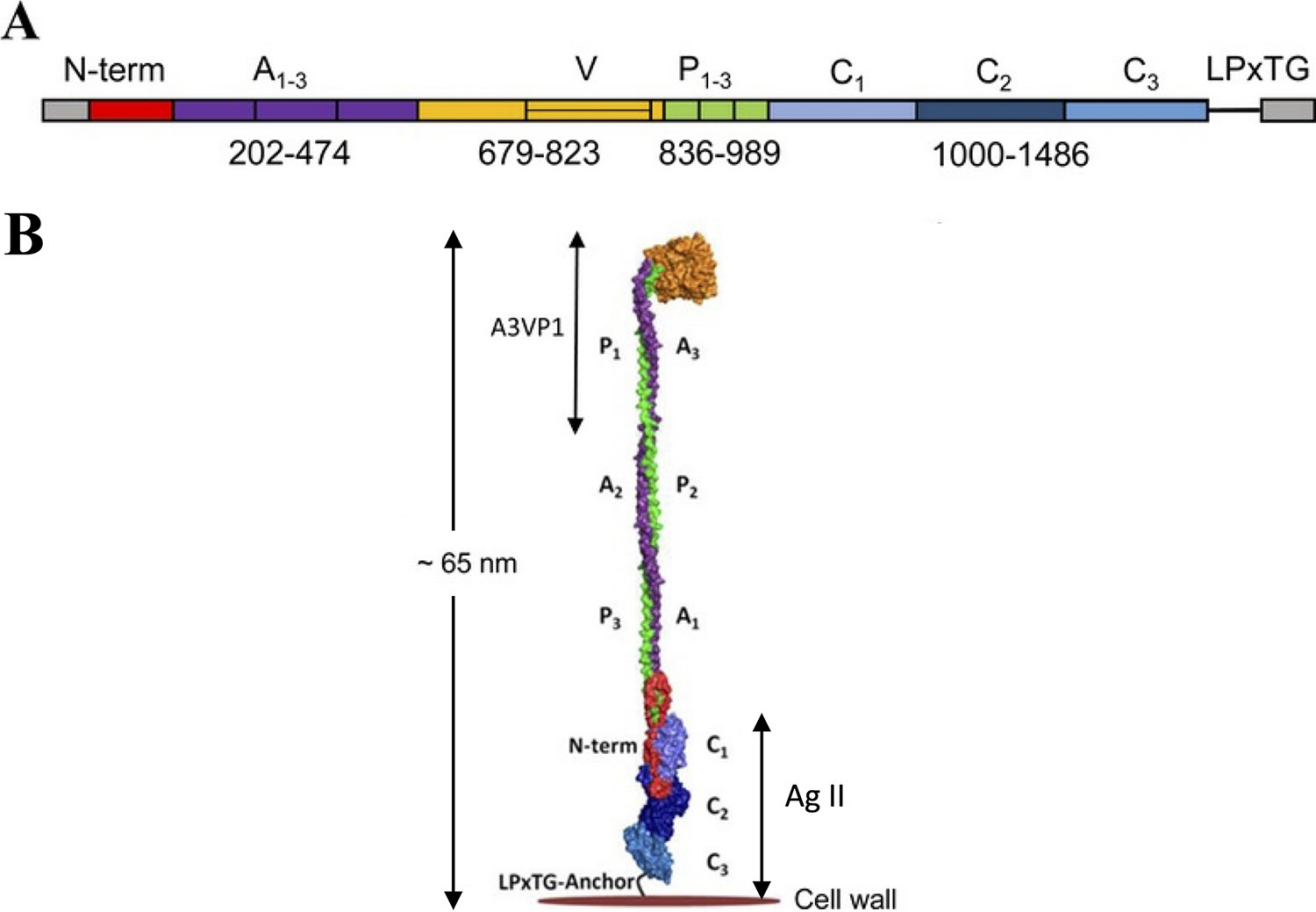
Schematic representation of P1(AgI/II) and relevant domains. (A) Schematic representation of relevant domains identified within the primary sequence of adhesin P1. A_1-3_, series of three alanine-rich tandem repeats; V, variable region where most sequence differences among strains are clustered; P_1-3_, series of three proline-rich tandem repeats; C1, C2, C3, beta-rich domains each of which adopts a DE-variant IgG fold; LPXTG, Sortase A cleavage motif. (B) Schematic representation of the tertiary structure of P1. The color scheme and indicated domains from the N to C terminus matches those shown in A. The protein folds into an unusual tertiary structure whereby the alanine-rich and proline-rich repeat regions interact to form an extended helical stalk. AgII, comprised largely of the C1 through C3 domains, not only is contained as an integral part of the cell-surface localized adhesin but also is liberated as an independent fragment capable of interacting with the globular head of the cell-surface localized adhesin.

As is typical for amyloidogenic polypeptides, structure prediction algorithms indicate that these four S. mutans proteins have considerable β-sheet structure (29, 30). P1, the sucrose-independent adhesin encoded by *spaP* and originally identified as Antigen I/II, is the most extensively characterized of these proteins (31). It exceeds 1,500 amino acids in length and contains 3 alanine-rich tandem repeats, a variable region where most strain-specific differences are clustered; a series of 3 proline-rich tandem repeats; and C-terminal membrane and wall spanning sequences, including an LPXTG sortase recognition motif (31, 32) (Fig. 1A). Crystal structures have been solved for two major segments, denoted A3VP1 and C123, enabling an almost complete elucidation of the protein tertiary structure (Fig. 1B). P1 folds into an unusual, elongated structure in which the alanine-rich and proline-rich repeat segments interact to form an extended helical stalk that projects a globular head at the apex (33). The amyloidogenic C-terminal region contains C1, C2, and C3 domains, with each having an extensive β-sheet structure (34). P1 also demonstrates a higher order quaternary structure in which these domains not only are present as an integral part of the full-length cell surface-localized adhesin but also are contained within the β sheet-rich carboxy-terminal AgII segment, which is liberated as an independent fragment that interacts with the P1 globular head to enhance bacterial adhesion (34–38). A recombinant C123 construct spanning C1 through C3 is standardly used as a tool in our laboratory to evaluate inter- and intramolecular interactions of P1, including amyloidogenesis (27, 30, 38).

In our previous work, we showed that mechanical agitation of S. mutans recombinant C123 or AgA or acidic pH treatment of recombinant Smu_63c induces amyloids that demonstrate a mat-like structure, in which the classic amyloid fibers are visible only after the mats are exhaustively digested with proteinase K to remove nonamyloid material (30). Amyloid mats and isolated fibers derived from purified C123, AgA, and Smu_63c all generated signature X-ray diffraction patterns characteristic of the classical stacked β-sheet amyloid structure. Mat-like structures were reconstituted when isolated fibers and protein monomers were coincubated without agitation, suggesting that S. mutans amyloids do not exist naturally as isolated fibers (30). This result is in contrast to those of Escherichia coli and Salmonella enterica serotype Typhi curli and tafi amyloids, which are visualized readily within biofilm environments as nests or clusters of individual fibers (39). Similar to S. mutans amyloids, images of mat-like amyloid structures have also been visualized for several other bacterial species (40–43). We also discovered in our prior studies that S. mutans amyloid formation is a biofilm-associated process that is impeded when *srtA* encoding the Sortase A transpeptidase is deleted, suggesting that a cell surface nucleation event may aid in initiating amyloidogenesis (28). Furthermore, amyloid formation by S. mutans was observed to occur late during biofilm development and maturation, namely, only after 60 h or more of incubation (30).

Irrespective of the microbial species, biofilm development is known to be a multistage process. It includes initial attachment where free-floating organisms land on a surface, firm attachment in which microbial cells gather and attach, maturation during which the microbial cells replicate and the biofilm achieves architectural elements characteristic of given organisms, and ultimately dispersion in which sections of biofilm detach and release free-floating microbes for further colonization (44–46). In the case of the S. mutans Cnm adhesin, it was found that when this collagen-binding adhesin transitioned to amyloid form, its ability to adhere to collagen-rich surfaces was significantly diminished (29). This finding has raised the possibility that amyloid formation by S. mutans may potentially provide a mechanism for modulating the adhesive function of certain proteins when adhesion is no longer needed.

In the present study, we aimed to expand our understanding of where and when amyloid formation occurs during the growth and maturation of S. mutans biofilms. To this end, we analyzed the reactivity of two separate biofilm fractions, namely, tightly adherent compared with nonadherent material, from both 1-day- and 5-day-old S. mutans biofilms, with the amyloidophilic dyes Congo red (CR) and Thioflavin T (ThT). We also performed confocal microscopy and immunostaining on similarly prepared samples using a panel of antibodies against P1 and WapA (27, 36, 47), including anti-P1 monoclonal antibodies shown here to discriminate between amyloid and nonamyloid forms of recombinant C123. Lastly, we evaluated whether the adhesive property of C123 with the known physiologic ligand of P1, salivary agglutinin (SAG) (48), was impacted following amyloid aggregation. Our results confirmed that amyloid formation is indeed a late event in the progression of the S. mutans biofilm and further demonstrated that amyloid material is present primarily within the nonadherent fraction of the older biofilm cultures. In addition, similar to the loss of adhesive activity of Cnm to collagen following amyloid aggregation (29), the ability of purified C123 to interact with the known substrate of S. mutans, namely, SAG, was significantly impaired when in amyloid compared with that in monomeric form. Taken together, the results of multiple complementary approaches are consistent with an emerging model in which the transition from protein monomers to amyloid aggregates provides a mechanism to constrain the adhesive function of proteins, such as P1, WapA, and Cnm, that is needed for early biofilm development, thereby facilitating detachment of aging S. mutans biofilms.

## RESULTS

### S. mutans amyloid-forming proteins influence macrocolony morphology.

Current models for evaluating factors that influence microbial biofilm development include submerged biofilms, floating pellicles, subaerial biofilms, and macrocolony biofilms (reviewed in reference 49). In macrocolony biofilm models, the microbial species under study are inoculated onto nutrient-containing agar plates that may include an indicator dye to better visualize morphological variation. Macrocolony morphology provides insight into variables that can influence the development of biofilm architecture. We compared the macrocolony morphology of an S. mutans serotype *c* strain, UA159, with that of an isogenic mutant devoid of the three known amyloidogenic proteins P1, WapA, and Smu_63c (27). UA159 does not encode the amyloidogenic protein Cnm. While glycosyltransferases (GTFs) contribute to S. mutans glucan production and biofilm formation (50), GTFs are not amyloidogenic (27). Therefore, as a negative control, we also included a UA159 mutant strain lacking genes encoding GtfBCD (27). In addition, we evaluated the macrocolony morphology of a ΔsrtA mutant (28), which cannot link P1 or WapA to the cell surface.

**FIG 2:**
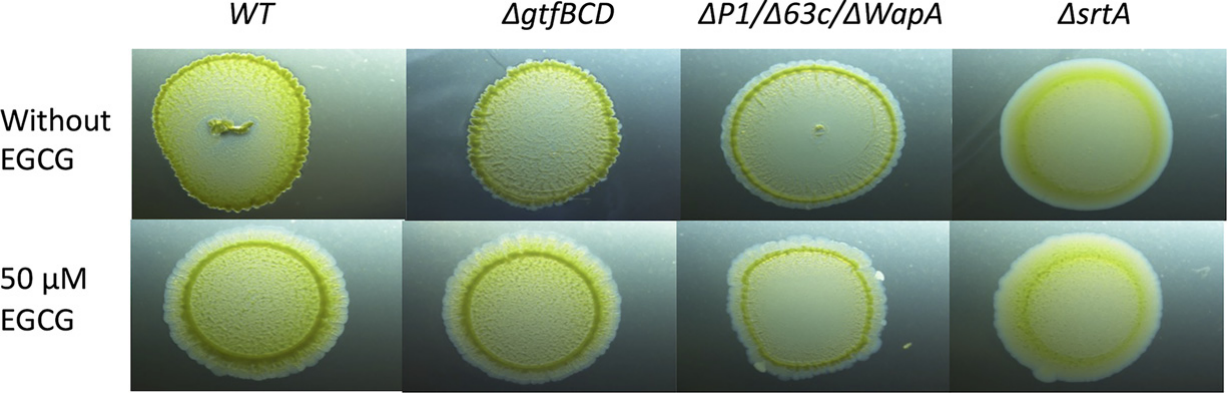
Evaluation of S. mutans macrocolony morphology. S. mutans wild-type UA159; a *ΔgtfBCD* mutant lacking glycosyltransferases B, C, and D; a *ΔspaP/wapA/smu_63c* mutant lacking amyloidogenic proteins P1, WapA, and Smu_63C; and a Δ*srtA* mutant lacking the Sortase A transpeptidase were spotted onto Todd-Hewitt plus yeast extract (THYE) agar plates containing Thioflavin S, with or without the amyloid inhibitor epigallocatechin gallate (EGCG). A halo-like morphology was observed for the *ΔspaP/wapA/smu_63c* mutant strain lacking known amyloidogenic proteins, for the Δ*srtA* mutant that cannot link substrate proteins to the cell surface, and for all four strains when grown in the presence of EGCG, suggesting that amyloid can influence S. mutans biofilm architecture.

When grown on Thioflavin S (ThS) indicator plates, macrocolonies of the wild type and Δ*gtfBCD* strains appeared similar. In contrast, the *ΔspaP/wapA/smu_63C* mutant displayed a “halo” effect that was absent from the other two strains (Fig. 2). The ΔsrtA mutant also displayed an outer halo, although its overall morphology was considerably smoother than the other strains. Such a result is not surprising given this mutant’s severely altered cell surface protein profile. When an inhibitor of amyloidogenesis, epigallocatechin gallate (EGCG), was incorporated into the agar, the halo morphology was observed for all four strains. The underlying mechanism of halo formation, which appears to be enhanced in the absence of amyloid, is unknown. However, observable differences in macrocolony morphology on the indicator plate imply that amyloid formation can influence S. mutans biofilm architecture.

### Localization of S. mutans amyloid formation using a submerged biofilm model.

S. mutans biofilm formation is assessed more conventionally using submerged biofilm models in which bacteria are grown on a hard surface that is bathed in a liquid nutrient medium. Previously, we performed a time course experiment in which submerged S. mutans biofilms were evaluated every 12 h for the development of Congo red birefringence indicative of amyloid formation, over a period of 1 week (30). In that study, biofilms were scraped from the bottom of the culture plate wells and suspended together with cellular and acellular contents present in the supernatant. The combined cellular and extracellular macromolecular material was then pelleted by centrifugation for analysis. In the current study, we analyzed separately the adherent and nonadherent fractions of S. mutans biofilms that were grown submerged for either 1 or 5 days. We utilized the following three complementary methods to detect amyloid material in the two fractions: Congo red birefringence, Thioflavin-T fluorescence assays, and confocal microscopy with immunostaining using a panel of differentially reactive antibodies against S. mutans amyloidogenic polypeptides.

### Congo red birefringence is detected in the nonadherent fraction of 5-day-old biofilms.

**FIG 3:**
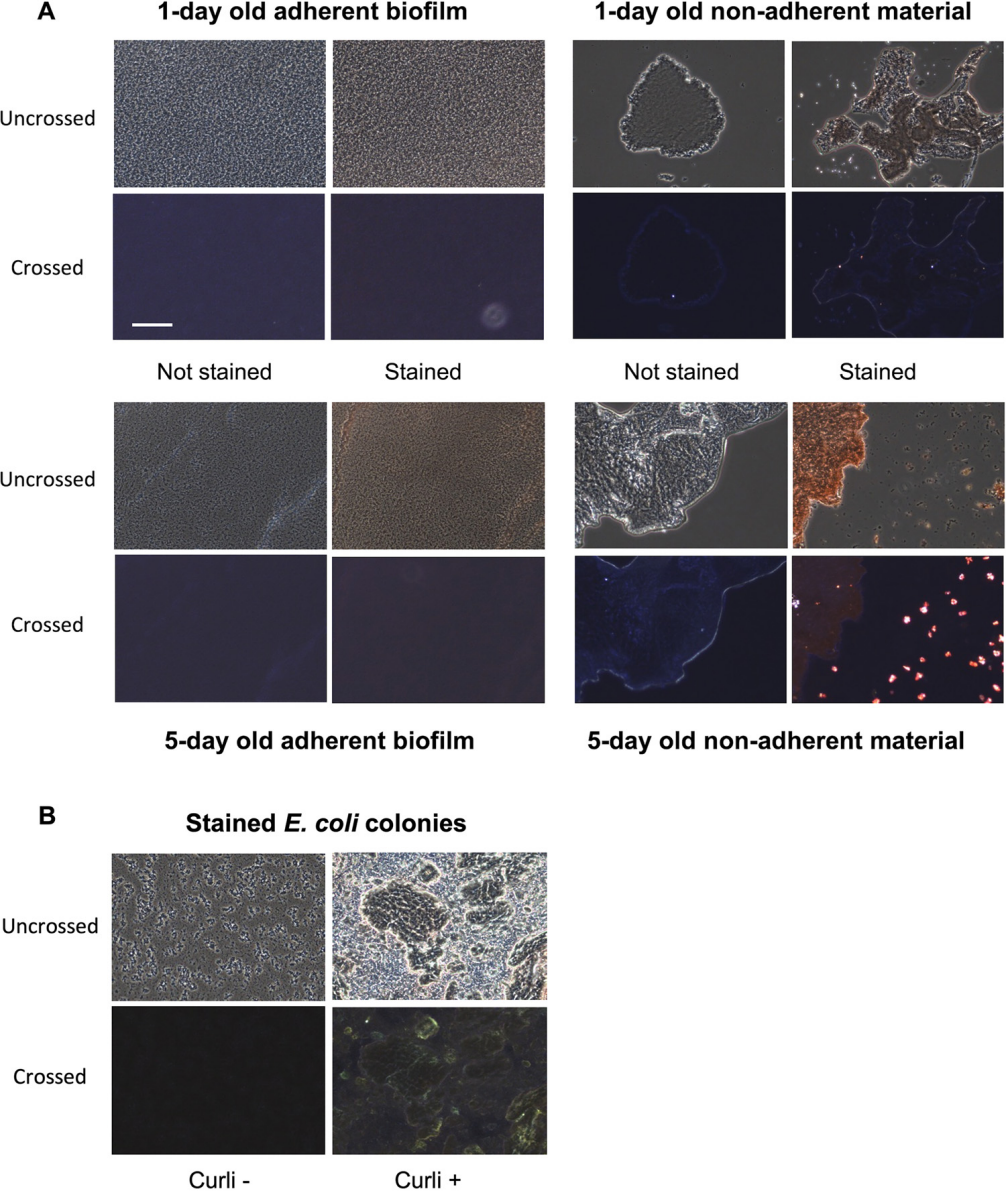
Evaluation of Congo red birefringence in adherent and nonadherent fractions of S. mutans biofilms. (A) Images of Congo red (CR)-stained adherent (left) and nonadherent (right) fractions from 1-day-old (top) or 5-day-old (bottom) biofilms viewed with and without crossed polarizers (“crossed” and “uncrossed,” respectively). (B) Images of CR-stained curli-negative (left) and curli-positive strains of Escherichia coli grown on YESCA agar and included here as negative and positive controls for CR birefringence. Scale bar: 115 μm (all panels).

The colors observed in the birefringence of Congo red (CR)-stained amyloid can vary from the classical “apple-green” to blue-green, yellow-green, yellow, orange, or red (51). Surprisingly, no birefringence was observed in tightly adherent 1- or 5-day-old biofilms that were stained with CR. In contrast, yellow-orange birefringent aggregates were observed when nonadherent material was removed from 5-day-old S. mutans biofilm cultures, stained with CR, and viewed through crossed polarizers (Fig. 3). Nonadherent material removed from 1-day-old biofilms did not demonstrate CR birefringence. CR birefringence was observed for Escherichia coli curli-positive cells grown on yeast extract/casamino acids (YESCA) agar which was used as a positive control. The corresponding curli-negative mutant did not demonstrate CR birefringence (52).

### Thioflavin-T fluorescence matches CR birefringence after bacterial cell wall material is eliminated from the samples.

While some studies have utilized *in situ* uptake and fluorescence intensity of the amyloidophilic dye ThT as a direct indicator of the presence of amyloid within growing S. mutans biofilms (53–55), ThT fluorescence can be misleading due to an interaction of ThT with nonamyloid components of the biofilms. Although amyloid formation is a biofilm-associated process for S. mutans (27, 28), we have shown previously that planktonically grown wild-type S. mutans, as well as mutants lacking P1, WapA, and Smu_63c, or Sortase A, all demonstrate an identical uptake of ThT (30). Therefore, in the absence of suitable controls and sample processing, ThT fluorescence of S. mutans biofilm cultures simply reflects cell growth.

**FIG 4:**
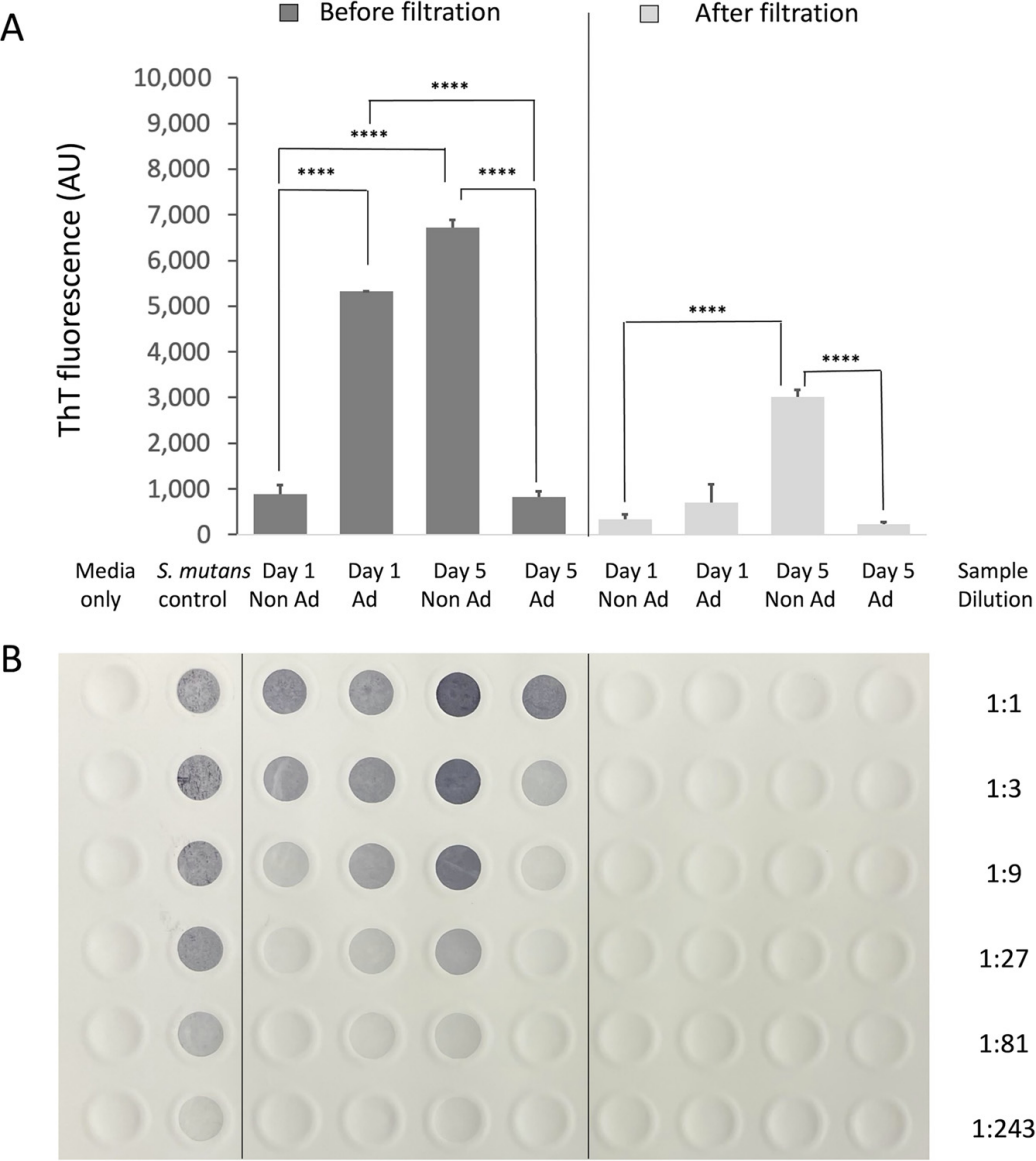
Thioflavin T fluorescence assay of adherent and nonadherent fractions of S. mutans biofilms before and after filtration. (A) ThT fluorescence assay of samples of adherent (Ad) or nonadherent (nonAd) material from 1- and 5-day-old S. mutans biofilms before and after filtration through a 0.2-μm filter. The experiment was performed in triplicate, with statistical analysis by one-way ANOVA (****, *P* < 0.0001). (B) Detection of residual cells or cell wall material in each sample by dot blot. Serial 3-fold dilutions were spotted onto the filter and probed with S. mutans serotype *c*-specific typing antiserum (CDC). A suspension of S. mutans from an overnight planktonic culture (beginning at ~3 × 10^7^ CFU), and growth media alone, served as positive and negative controls for the antiserum, respectively.

To overcome this limitation, we filtered adherent and nonadherent fractions derived from 1-day- and 5-day-old biofilms to remove whole bacterial cells or large cell wall fragments associated with autolysis in aging biofilms. When the filtered samples were analyzed, results of ThT fluorescence assays mirrored those of the CR-induced birefringence experiment; amyloidophilic dye-reactive material was localized to the 5-day-old nonadherent fraction (Fig. 4). We identified the presence of bacterial cells and/or cell walls in unfiltered and filtered samples using a dot blot probed with type-specific serotype *c* antiserum (Fig. 4B) (56). Prior to filtration, strong ThT fluorescence was observed in all samples, particularly those with the strongest anti-serotype *c* antibody reactivity (Fig. 4A). After filtration, no anti-serotype *c* reactivity remained in the filtrate (Fig. 4B), and yet, the ThT signal associated with the 5-day-old nonadherent fraction was significantly increased compared with that of the other samples (*P* < 0.0001).

### Characterization of anti-C123 antibodies used for immunostaining of S. mutans biofilm material.

**FIG 5:**
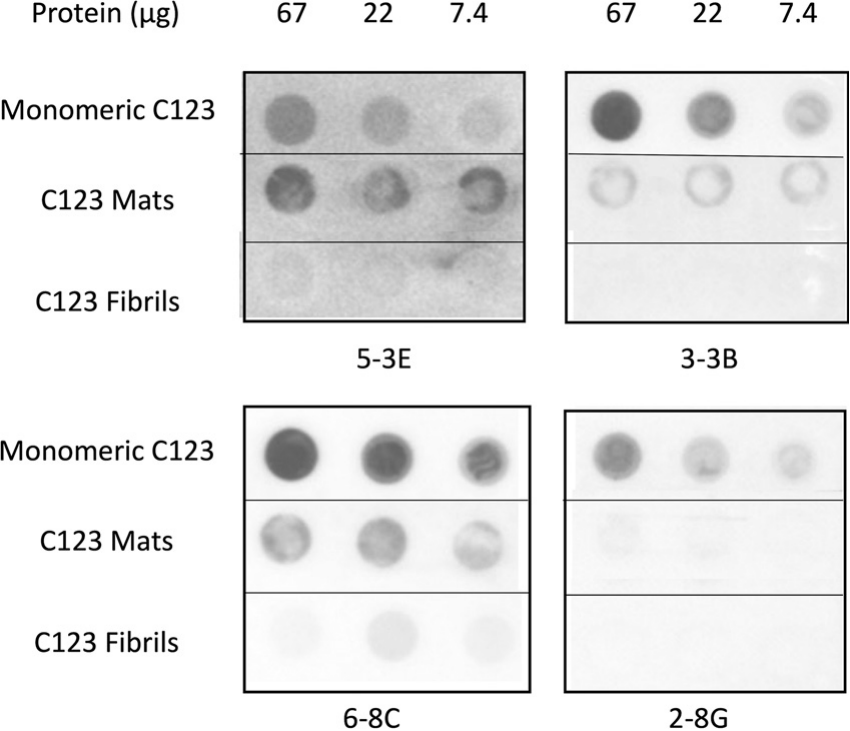
Evaluation of reactivity of anti-P1 monoclonal antibodies with monomeric, amyloid mat, and purified fibril forms of C123 by dot blot. The indicated amount of the total protein of monomeric C123, amyloid mats induced by mechanical agitation, or purified amyloid fibrils derived by proteinase K treatment of amyloid mats were spotted onto the filter and probed with the indicated anti-P1 MAb.

A panel of monoclonal antibodies was made previously against S. mutans adhesin P1 (57), and their approximate binding locations, cross-reactivity patterns, and biological activities were characterized in multiple studies (36, 47, 58, 59). In addition to monoclonal antibody (MAb) 4-10A, which recognizes an epitope repeated 3 times within the hybrid helical stalk of full-length P1, several different MAbs that recognize epitopes within C123 are also available (see Fig. S1 in the supplemental material). Recombinant C123 corresponds to the majority of the naturally occurring C-terminal P1 truncation product originally identified in spent culture supernatants as AgII (60). Approximate binding locations of C123-reactive MAbs 6-8C, 3-3B, 5-3E, and 2-8G were refined by measuring their reactivity by enzyme-linked immunosorbent assay (ELISA) against purified recombinant polypeptides corresponding to C1, C2, C3, C12, C23, and C123 (34) (see Fig. S2 in the supplemental material). While all four MAbs recognize intact C123, their epitopes differ based on variations in their functional behavior and reactivity profiles (31, 36, 47, 57, 59, 61). MAb 6-8C is a reagent commonly used in our laboratory that had been observed to be less reactive with mechanically induced C123 amyloid than the monomeric form of the protein. We therefore used a dot blot assay to systematically compare the reactivity of each of the C123-reactive MAbs with similar concentrations of the C123 monomeric protein, C123 amyloid-containing mats induced by mechanical agitation of the protein monomer, and C123 amyloid fibrils derived by exhaustive proteinase K digestion of the C123 mats to eliminate nonamyloid material from the mat ultrastructure (Fig. 5). As expected, MAb 6-8C showed diminished reactivity with C123 amyloid mats compared with the monomer. This effect was somewhat more pronounced for MAb 3-3B and was also observed for 5-3E. Little to no reactivity was observed with isolated amyloid fibrils for any of the C123-reactive MAbs. Among the four MAbs tested, 2-8G was unusual in that it was essentially nonreactive with the amyloid mat form of C123. Therefore, a comparison of degrees of immunoreactivity of 2-8G and 6-8C, which represents the pair of MAbs most and least impacted by amyloid induction, is an appropriate tool for discriminating amyloid from nonamyloid forms of the AgII truncation derivative of P1 *in situ* within S. mutans biofilms.

### Confocal microscopy and immunostaining of adherent and nonadherent fractions of S. mutans biofilms.

Following a similar design as described above for CR birefringence and ThT uptake experiments, we evaluated adherent and nonadherent fractions of 1- and 5-day-old S. mutans biofilms by confocal microscopy and immunostaining using antibodies against P1 and WapA. We utilized the aforementioned 4-10A, 6-8C, and 2-8G murine anti-P1 MAbs, as well as a polyclonal rabbit antiserum specific for AgA, the amyloidogenic truncation derivative of WapA (27). A green fluorescent protein (*gfp*)-expressing strain of S. mutans UA159 was used for confocal microscopy imaging.

**FIG 6:**
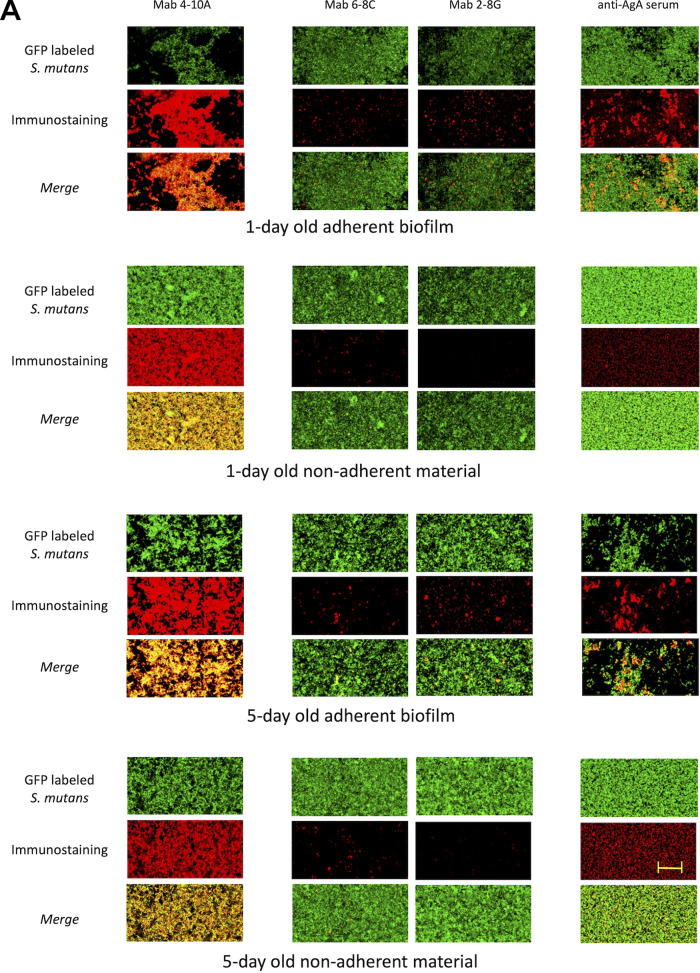

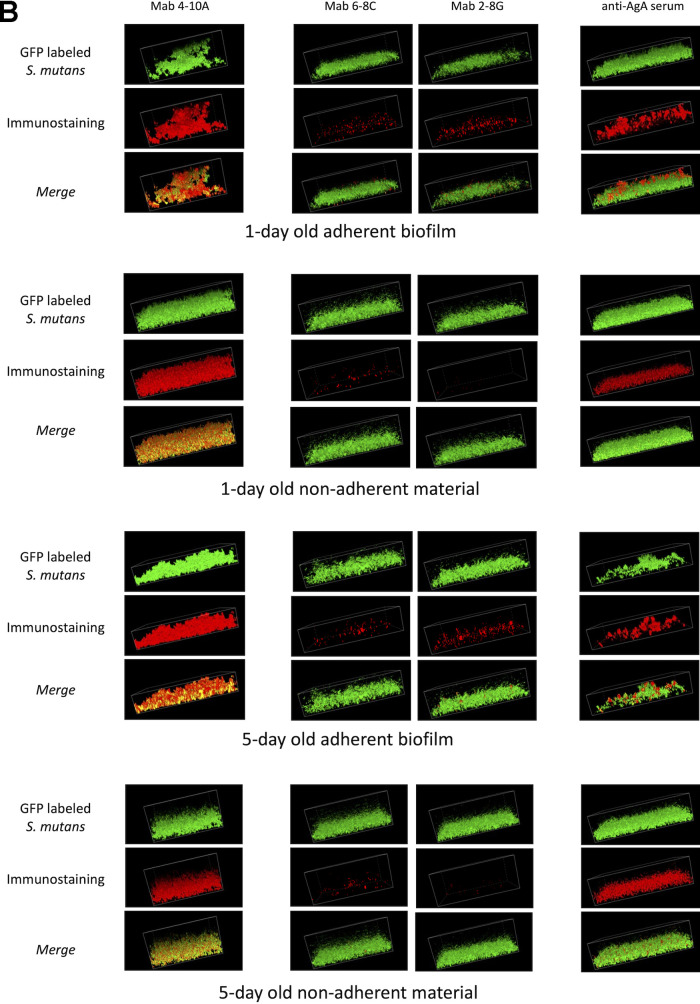
Confocal microscopy and immunostaining of adherent and nonadherent fractions of S. mutans biofilms. S. mutans (UA159*::Pldh-gfp*) biofilms were grown for 1 or 5 days and then immunostained with the indicated antibody. Adherent fractions were stained directly on the slide on which the biofilm was grown. Nonadherent fractions were transferred to a tube and stained separately. Goat anti-mouse or anti-rabbit secondary reagents were conjugated to Alexa Fluor 594 (red). (A) Maximum intensity projections. Scale bar: 20 μm. (B) Volume snapshots corresponding to images shown in A.

As shown in maximum intensity projections (Fig. 6A), there was strong concordance between MAb 4-10A staining and cellular GFP fluorescence in both 1- and 5-day adherent biofilms. MAb 4-10A was also strongly reactive with the cellular material present in the nonadherent fractions. This finding indicates that 4-10A recognizes primarily full-length P1 that is covalently attached to the cell wall peptidoglycan. In contrast, the reactivity of C123-reactive MAbs 6-8C and 2-8G appeared extracellular and did not colocalize with the GFP-labeled cells. Of particular interest was the relative diminution of 2-8G reactivity compared with that of 6-8C in the nonadherent fractions. The results strongly suggest that the AgII derivative of P1 is present in both the adherent and nonadherent fractions of S. mutans biofilms but that a monomeric form predominates in the tightly adherent layer while an aggregated form is dominant in the nonadherent material. The ratio of 2-8G, but not 6-8C, immunofluoresence/GFP green fluorescence was significantly decreased in the nonadherent biofilm fractions (see Fig. S3A in the supplemental material). The relative loss of 2-8G compared with 6-8C staining is consistent with the detection of amyloid primarily in the 5-day-old nonadherent fraction by both CR-induced birefringence and ThT fluorescence (Fig. 3 and 4).

To determine whether the cellular component of the amyloid-containing nonadherent biofilm fraction reflected a population of cells that had adhered and detached, or never attached in the first place, another 2-8G/6-8C immunostaining experiment was performed. In this case, biofilms were grown for 3 days, then all nonadherent material was removed, biofilms were washed, fresh growth medium was added, and biofilms were incubated for an additional 3 days. Again, the ratio of 2-8G, but not 6-8C, immunofluoresence/GFP green fluorescence was significantly decreased in the nonadherent biofilm fraction (Fig. S3B).

Unlike P1, for which a panel of monoclonal antibodies is available, the only reagent currently available for detecting AgA is a monospecific polyclonal antiserum. Nevertheless, these results were also highly informative. Immunostaining of adherent biofilms with anti-AgA antiserum was patchy in nature and, unlike anti-P1 MAb 4-10A, did not colocalize with the cellular material. In addition, the relative amount of AgA detectable in the nonadherent fractions appeared to increase as the biofilms aged. No reactivity of anti-mouse or anti-rabbit secondary reagents alone with adherent or nonadherent material was observed in these experiments (see Fig. S4 in the supplemental material). We also evaluated three-dimensional (3D) volume snapshots of the same confocal images to enhance our understanding of where the polypeptides were localized throughout the depth of the biofilm (Fig. 6B). As also seen in Fig. 6A, we observed 4-10A staining to be associated closely with GFP-labeled cells. In contrast, binding of antibodies against C123 and AgA was patchy and exclusively extracellular. While clusters of anti-AgA reactive material lay primarily on top of the 1-day-old adherent biofilm, this pattern changed in the 5-day adherent biofilm in which AgA clusters appeared to penetrate throughout the biofilm. In addition, the amount of extracellular AgA in the nonadherent fraction appeared to be increased in the 5-day compared with that in the 1-day old sample, with smaller clusters than the adherent samples.

### Amyloid aggregation diminishes the adhesin activity of C123 with human salivary agglutinin.

**FIG 7:**
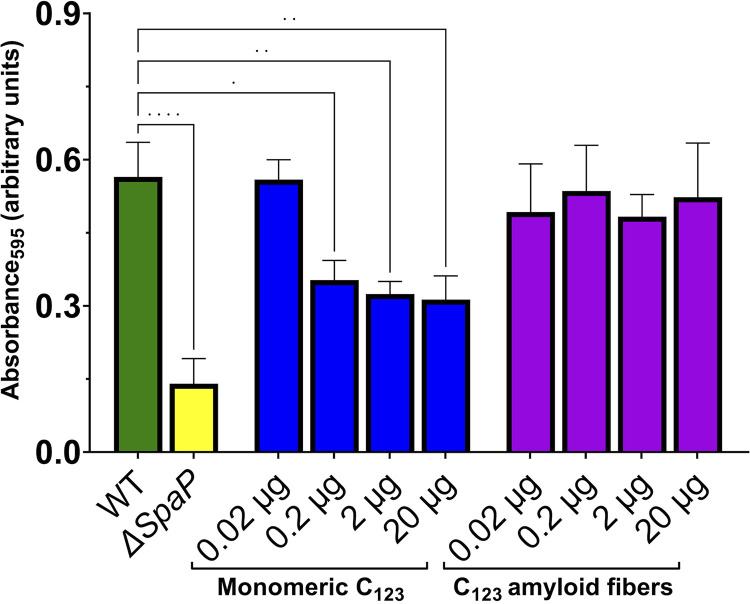
Competitive inhibition of S. mutans binding to immobilized salivary agglutinin by C123 monomers compared with that by C123 amyloid fibrils. Microtiter plate wells were coated with human salivary agglutinin and then incubated with the indicated amount of C123 monomers or purified amyloid fibrils. Following washing, ~3.5 × 10^8^ CFU of S. mutans was added to the wells and incubated for 3 h, cell attachment was assayed by staining with crystal violet, and measurement of absorbance at 595 nm was conducted. Wild-type S. mutans in the absence of an added inhibitor and a *ΔspaP* mutant lacking the gene encoding adhesin P1 were included as positive and negative controls, respectively. The experiment was performed in triplicate, with statistical analysis by one-way ANOVA. ns, not significant; *, *P* < 0.05; **, *P* < 0.01; ***, *P* < 0.001; ****, *P* < 0.0001.

Because anti-C123 reactive material was associated primarily with the nonadherent, rather than tightly adherent, component of S. mutans biofilms, we tested whether the adhesive function of C123 with the physiologic binding partner of P1 was diminished during amyloid aggregation. P1 and AgI/II homologs of other oral streptococci have long been known to interact with salivary agglutinin (SAG), a high-molecular-weight complex that is immobilized on the tooth surface as part of the salivary pellicle (48). SAG is comprised of the scavenger receptor cysteine-rich (SRCR) glycoprotein gp340, secretory IgA, and an 80-kDa protein (62). In fluid phase, gp340 is an important mediator of innate immunity that is also known as DMBT1 (63, 64). We compared the ability of C123 amyloid fibrils and C123 monomeric protein, over a similar range of concentrations, to competitively inhibit the adherence of S. mutans to SAG immobilized on a solid surface (Fig. 7). In contrast to the wild-type parent strain, the *ΔspaP* mutant strain devoid of P1 was severely impaired in its ability to adhere to the immobilized SAG (*P *< 0.0001). In the presence of the added C123 monomer, adherence of wild-type S. mutans was significantly inhibited (*P *< 0.01), indicating that the purified protein was able to compete for binding to the immobilized SAG. Previous studies have shown that there are two discrete SAG binding sites within P1 and that competition from the C123 fragment monomer alone can inhibit the binding of S. mutans to immobilized SAG by as much as 40% (33, 34, 48). Unlike monomeric C123, however, comparable concentrations of C123 amyloid fibrils showed no ability to interfere with the binding of S. mutans to immobilized SAG since no significant diminution of bacterial adherence was observed (Fig. 7). This loss of competition ability indicates that amyloid aggregation diminishes the SAG-adhesive function of C123.

## DISCUSSION

Microorganisms utilize amyloid aggregation for a variety of functional purposes (15–24, 65). Our current work is consistent with a model in which amyloid formation by S. mutans proteins facilitates the detachment of older biofilms. Four complementary lines of evidence support this conclusion. First, CR birefringence was observed only in the nonadherent fraction of older (5 day) biofilms. Second, once nonamyloid-related background reactivity stemming from the presence of cell wall material was eliminated by filtration of biofilm samples, ThT fluorescence assays also identified amyloid material only in the nonadherent fraction of the older biofilm. Although other studies have reported, based on ThT uptake alone, that amyloid formation is an early event associated with adherent S. mutans biofilms (53–55), our more rigidly controlled experiments indicate otherwise. The third line of evidence comes from confocal microscopy and immunostaining with two different C123-reactive monoclonal antibodies that can discriminate between amyloid and nonamyloid forms of the polypeptide. Again, results were consistent with the presence of amyloid only within the nonadherent component of our biofilm samples. Similar results were obtained when tightly adherent biofilms were washed and incubated in fresh media to confirm that cellular and extracellular material in the nonadherent fractions represented detached biofilm. The fourth line of evidence comes from a competitive binding assay that showed that the adhesive function of the C123 fragment of P1 is significantly impaired following amyloid aggregation. Monomeric C123, but not C123 amyloid fibers, competitively inhibited binding of S. mutans to its physiologic substrate, immobilized SAG. This result is the second reported instance of such a finding. Amyloid aggregation of Cnm, which is produced by a subset of S. mutans strains associated with endocarditis (66), abrogated the ability of this adhesin to competitively inhibit the adherence of strain OMZ175 to immobilized collagen (29). Thus, amyloid aggregation by multiple S. mutans adhesins appears to represent a regulatory mechanism in which their adhesive activity is dampened when no longer necessary thereby facilitating biofilm detachment.

A new paradigm is emerging for Gram-positive bacteria, in which certain cell surface adhesins perform dual functions, with the second being amyloid aggregation (23, 41, 67). Often, such adhesins, including biofilm-associated protein (Bap) of Staphylococcus aureus, enterococcal surface protein (Esp) of Enterococcus faecalis, and P1 and WapA of Streptococcus mutans, are processed to truncated derivatives that serve as the building blocks for the amyloid aggregates (27, 28, 41, 42). It is coming to light that such aggregation is influenced by prevailing environmental conditions, for example pH, surface hydrophobicity, and/or divalent cation concentrations (23, 27, 42, 43, 68, 69). The environmental triggers of S. mutans amyloid formation are not yet known and are the focus of continued study. A number of amyloidogenic proteins produced by Gram-positive bacteria link their full-length cell-associated forms to the cell wall peptidoglycan via a sortase transpeptidase enzyme (14, 27, 28, 41, 42). In Gram-negative bacteria, such as Escherichia coli and Salmonella Typhi, the production of curli and tafi fibers is a two-stage process in which a nucleator protein, CsgB, is tethered to the bacterial surface, while the extracellular CsgA component associates with CsgB to begin the process of elongation of CsgB amyloid fibers (70–72). In the case of some Gram-positive bacterial amyloids, it now appears that these two roles may be accomplished by the same parent polypeptide. One form is covalently attached to the cell wall but can also interact with a truncated and/or extracellular derivative that under appropriate conditions within the biofilm undergoes the process of amyloid fibrillization.

In the current study, we evaluated two S. mutans Sortase A-linked adhesins, whose respective truncation derivatives are amyloidogenic, to determine where and when the polypeptides are found in relation to amyloid during the course of biofilm progression. S. mutans P1 has been characterized more extensively than WapA at both structural and functional levels; however, it is known that WapA is similar to the amyloidogenic adhesin Cnm in that both are posttranslationally modified and glycosylated by the same PgfS glycosyltransferase (73). In contrast to P1, crystal structures are not yet available for WapA or Cnm. Before elucidation of its tertiary structure (34, 35, 74), it was thought that MAbs reactive with the P1 C terminus lacked reactivity with whole S. mutans cells because their epitopes were inaccessible when the protein was linked to the cell wall peptidoglycan (61). It was a mystery, however, how such MAbs could also be strong inhibitors of S. mutans adherence to salivary agglutinin (58). This paradox was explained when the quaternary structure of P1 became better understood. The C terminus of P1 is present in two places at once. Recombinant C123, which comprises almost all of AgII, was shown to bind specifically to the apical head of cell wall-attached P1 (Fig. 1B) (37, 38). An antibody-triggered release of P1 fragments from the cell surface had earlier been postulated as a potential mechanism of S. mutans immune evasion (75). This speculation was confirmed when glutaraldehyde-fixed and nonfixed S. mutans cells were incubated with C123-reactive MAbs (36). Binding of such MAbs to unfixed cells triggered the release of the 60-kDa AgII fragment of P1 from the bacterial cells, while fixation resulted in an increased detection of their reactivity (36).

While there appears to be substantially greater reactivity in Fig. 6 of the 4-10A anti-P1 MAb and polyclonal anti-AgA rabbit antiserum than that of MAbs 6-8C and 2-8G, this finding is likely due to a relative loss of AgII and associated antibodies during washing steps. We opted not to use glutaraldehyde fixation in the current study because we wished to be able to discern amyloid localization within adherent compared with nonadherent fractions within the biofilm. More important than the relative reactivity of the anti-C123 MAbs compared to 4-10A and anti-WapA reagents is the relative degrees of reactivity of MAbs 6-8C and 2-8G with amyloid and nonamyloid forms of the protein. These results were congruent with CR birefringence and ThT uptake of filtered samples, which also localized amyloid to the nonadherent biofilm fraction.

**FIG 8:**
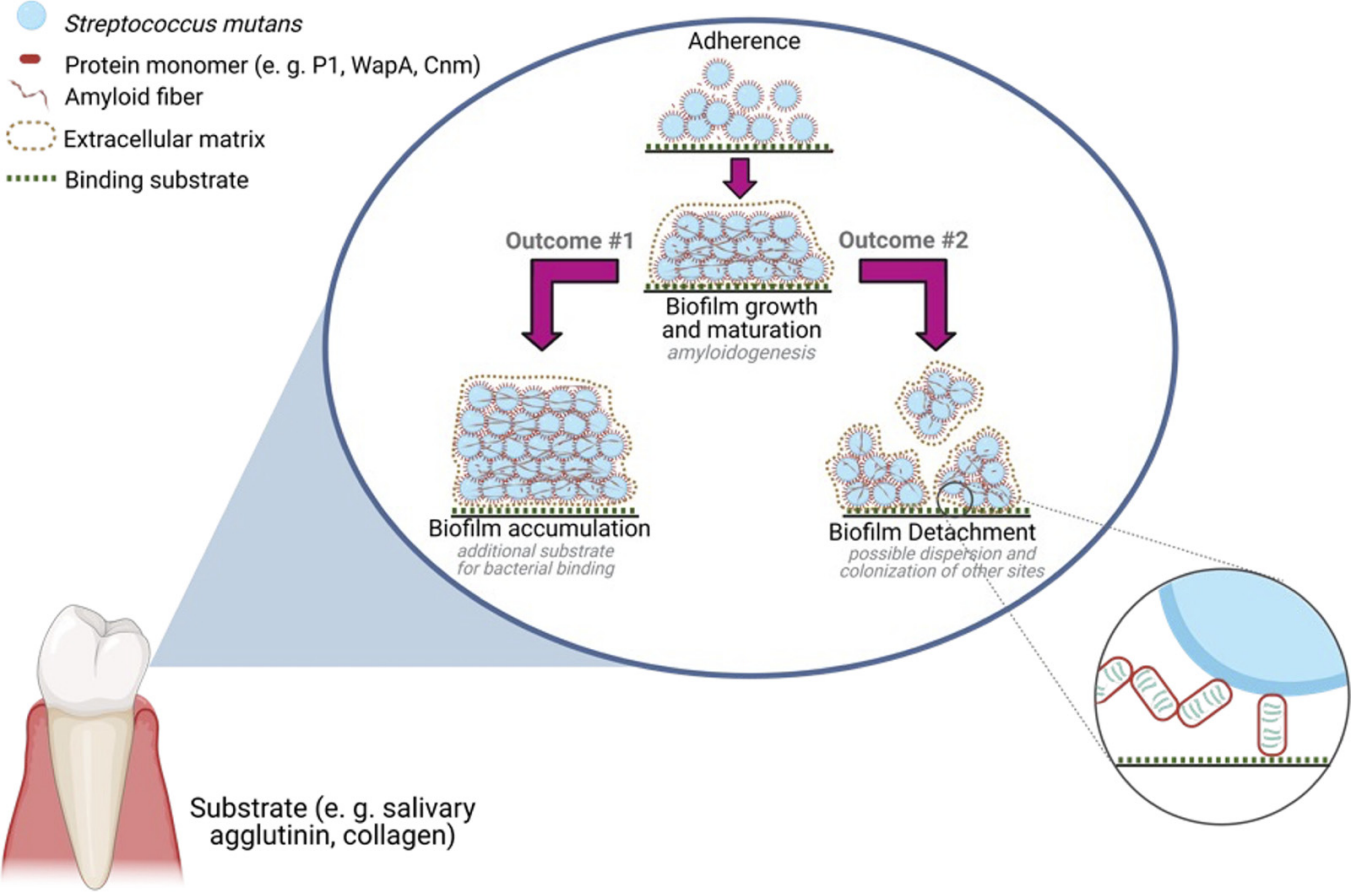
Working model for amyloid-induced detachment of S. mutans mature biofilms. Initially, monomeric cell surface-anchored adhesins are available for binding to their substrates, allowing for initial colonization and biofilm development. As the biofilm grows and matures, amyloidogenic moieties associated with the cell surface or in the extracellular matrix undergo amyloid aggregation, which can either act as a scaffold for the development of a robust adherent biofilm layer (outcome 1) or can disrupt adhesive activity and promote biofilm detachment (outcome 2). Created with BioRender.com.

Rather than nests of individual amyloid fibers that are observed within E. coli and S. Typhi biofilms (39), amyloid-containing extracellular matrices produced by Gram-positive species appear more like sheets or mats of aggregated material (27, 30, 40–43). Such extracellular matrices also contain polysaccharides and extracellular DNA whose interaction with amyloid and amyloidogenic proteins is a growing topic of study (76–78). It has been speculated that amyloid serves as a tool for mediating intercellular interactions characteristic of biofilm communities (23). Indeed, immunogold staining for P1 and AgA within S. mutans biofilms revealed both to be contained largely within extracellular matrices that connected cells to one another (27). Our current study asked whether amyloid formation within growing and maturing S. mutans biofilms promotes cellular accumulation on the underlying surface or alternatively contributes to cellular and extracellular matrix interactions that facilitate biofilm detachment (Fig. 8). Our results support the latter scenario. Hence, a new model is emerging in which amyloid aggregation appears to serve as a mechanism to temper the adhesive activity of the component polypeptides, facilitating the detachment of cells and extracellular material from aging biofilms. As the biofilms age, CR-induced birefringence and staining with anti-C123 and anti-AgA antibodies appear patchy and punctate in nature and are not congruent with the more tightly adherent layer. In addition, amyloid aggregation of two different proteins, C123 and Cnm, abrogates their ability to competitively inhibit the adhesion of S. mutans to their cognate substrates, namely, immobilized SAG and collagen, respectively. Thus, dampening of the primary adhesive function of S. mutans adhesins following their amyloid aggregation may represent a conserved mechanism to facilitate the detachment and dissemination of biofilm material from a solid surface. Whether this finding represents a more widespread property of Gram-positive amyloidogenic proteins will be of interest in future studies.

## MATERIALS AND METHODS

### Bacterial strains and growth conditions.

S. mutans strains UA159 and NG8 were used in this study. For confocal microscopy and immunostaining, S. mutans producing green fluorescent protein (GFP) (UA159::*Pldh-gfp*) (79) was used. For S. mutans biofilms, overnight cultures grown in Todd-Hewitt broth (THB; Difco) were diluted 20-fold in biofilm media (BM) (80) containing 20 mM glucose (BMG) and grown to an optical density at 600 nm (OD_600_) of ~0.2 to 0.4. Cultures were then diluted to an OD_600_ of 0.05 in BMG and placed into the wells of chamber slides (ibidi GmbH, catalog [cat.] number 81816 for immunostaining [120 μL]; cat. number 80827 for CR staining [300 μL]; or a 12-well tissue culture plate [Costar] for ThT assay [1.2 mL]). Slides/plates were incubated in 5% CO_2_ at 37°C for 1 or 5 days, at which time nonadherent material was pipetted gently from the tightly adherent biofilm layer. For immunostaining of the 5-day biofilm cultures, 20 μL of BMG was added per well on days 2 and 4. Tightly adherent biofilms and nonadherent material were pelleted by centrifugation and evaluated in separate experiments as described below by immunostaining/confocal microscopy, Congo red (CR), and Thioflavin T (ThT) staining and dot blot analysis.

### Macrocolony morphology.

Macrocolony morphology was assessed using a modification of the method described by Serra et al. (81). S. mutans strains were grown overnight in Todd-Hewitt broth, and 5 μL of the culture was spotted onto agar plates containing chemically defined medium (82) and 40 μg/mL Thioflavin S dye, with and without the addition of 50 mM the amyloid inhibitor epigallocatechin gallate. Plates were incubated at 37°C in 5% CO_2_ for 5 days, and macrocolony morphology was visualized using a Nikon SMZ 745T stereomicroscope (10× magnification) integrated with a Nikon DS-Vi1 digital camera.

### Congo red birefringence.

Adherent biofilms and corresponding nonadherent samples were stained with CR before imaging as described in reference 28. Stained material was visualized with a Nikon Eclipse Ts2R inverted microscope equipped with two polarizers using a 10× objective. Images were viewed with and without crossed polarizers, which reveals CR birefringence compared with total material present, respectively. CR-stained curli-negative and curli-positive strains of Escherichia coli grown on YESCA agar (52) were included as positive and negative controls, respectively, for CR staining and birefringence.

### Thioflavin T uptake before and after elimination of residual cells and cell wall material.

A 9.1 mM stock solution of ThT (Fluka) was prepared in pure H_2_O. The stock concentration was determined by measurement of absorbance at 412 nm using the extinction coefficient ε_412_ of 31,600 M^−1^cm^−1^ (83). After 1 or 5 days of biofilm growth, spent liquid medium containing nonadherent material from six culture plate wells was carefully removed, combined, and adjusted to a volume of 7.2 mL with BM without glucose. Fresh BM without glucose was added to the emptied wells (1.2 mL/well), and adherent material was scraped off and suspended in the medium. Half of the 7.2 mL of resuspended sample was filtered through a 0.22-mm acrodisc (Nalgene) to eliminate bacterial cells or large fragments of cell walls stemming from the autolysis of aging biofilms. Twenty-five microliters of each nonfiltered or filtered sample and 25 μL of 4 μM ThT in H_2_O were added to the wells of a 96-well microplate (Corning) and incubated in the dark with gentle rocking for 1 h at room temperature. Fluorescence intensity was measured using a BioTek Synergy H1 spectrophotometer at 440-nm excitation and 485-nm emission wavelengths, with the assay performed in triplicate. Two hundred microliters of 3-fold serial dilutions of the nonfiltered or filtered sample was also applied to a prewet nitrocellulose membrane using a dot blot manifold (MiniFold I; Whatman). The filter was blocked with phosphate-buffered saline (PBS) containing 0.03% Tween 20 and 5% skim milk, probed for the presence of contaminating cell wall material using anti-S. mutans typing antiserum (CDC) (1:500) followed by horseradish peroxidase (HRP)-labeled goat anti-rabbit IgG (MP Biomedicals) (1:833) and developed with 4-chloro-1-naphthol substrate solution as described previously (58). Two hundred microliters of a suspension of S. mutans cells from an overnight culture in Todd-Hewitt broth (THB), beginning at ~3 × 10^7^ CFU/mL, was used as a positive control to confirm the reactivity of the anti-serotype *c* antiserum. THB medium only was used as a negative control.

### Reactivity of anti-P1 monoclonal antibodies with recombinant C-terminal polypeptides.

Microtiter plates were coated overnight at 4°C with 100 mL of 0.1 M carbonate-bicarbonate buffer containing 0.02% sodium azide and 0.2 mg of purified recombinant C1, C2, C3, C12, C23, or C123 polypeptide (kind gifts from Champion Deivanyagam, University of Alabama) (34). Plates were washed with PBS and blocked with PBS containing 0.3% Tween20 (PBST). Coated wells were incubated with 3-fold serial dilutions (in PBST) beginning at 1:50 of anti-P1 MAbs 6-8C, 3-3B, or 2-8G (61) washed; incubated with HRP-labeled goat anti-mouse IgG (Cappel) (1:1,000 in PBST); and washed and developed with a *o*-phenylenediamine dihydrochloride substrate solution. Plates were read using a Bio-Rad iMark plate reader using MPM 6.0 software, and absorbance was measured at 450 nm.

### Dot blot assay to evaluate the reactivity of anti-P1 MAbs with monomeric, amyloid mat, and amyloid fiber forms of purified recombinant C123.

Recombinant C123 was purified as described previously (30). Induction of the C123 amyloid by mechanical agitation and isolation of purified C123 amyloid fibers by exhaustive digestion of induced amyloid mats with proteinase K were also performed as described previously (30). Three-fold serial dilutions of each sample were prepared in 50 mM sodium phosphate containing 100 mM NaCl (pH 8.0), with 200-μL aliquots of each dilution applied using a dot blot manifold (MiniFold I; Whatman) to a prewet polyvinylidene difluoride (PVDF) membrane presoaked in 100% ethanol. The membrane was blocked with PBS containing 0.05% Tween 20 and 5% skim milk. Replicate blots were reacted with anti-P1 monoclonal antibodies 6-8C, 3-3B, or 2-8G diluted 1:500 in blocking buffer; washed; and reacted with HRP-conjugated goat anti-mouse IgG (1:500) (MP Biomedicals). After the wash step, antibody reactivity was traced using a chemiluminescent detection kit according to the manufacturer’s instructions (Amersham ECL prime Western blotting detection reagent; GE Healthcare).

### Immunostaining and confocal microscopy.

Spent medium containing nonadherent cellular and extracellular material was carefully removed from 1- and 5-day-old biofilm cultures, transferred to 0.2-mL PCR tubes, and centrifuged at 22°C for 5 min at 16,100 × *g*. The supernatants were removed and pellets resuspended in 150 μL PBS containing 5% of heat-inactivated qualified fetal bovine serum (Thermo Fisher Sci.) (PBS-FBS). A total of 150 mL of PBS-FBS was added to adherent biofilm layers remaining on the slide. All samples were incubated at room temperature for 1 h with gentle rocking. Following the blocking step, samples were incubated for 2 h at room temperature with 100 μL primary antibodies diluted in PBS-FBS. They included anti-P1 monoclonal antibodies 4-10A, 6-8C, and 2-8G and anti-AgA rabbit antiserum that had been exhaustively adsorbed against a S. mutans *ΔwapA* mutant to remove cross-reactivity (27). Primary antibody dilutions were: 1:1,000 for 4-10A, 1:400 for 6-8C and 2-8G, and 1:250 for anti-AgA. Samples were washed with PBS then reacted with 100 μL of diluted in PBS-FBS Alexa Fluor 594-labeled goat anti-mouse (1:500) or anti-rabbit (1:1000) cross-adsorbed secondary antibodies as appropriate (Invitrogen). After incubation for 1 h at room temperature, samples were maintained at 4°C overnight. The following day, samples were warmed to room temperature for 30 min and washed with PBS, and 120 μL of PBS was added to each sample. Nonadherent samples stained in tubes were resuspended by vortexing and then were transferred to clean wells of an 18-well ibidi slide and allowed to settle for at least 3 h before imaging. Confocal microscopy images of adherent and nonadherent samples were collected using a Nikon Eclipse Ti2 inverted confocal microscope (60× objective, 0.1 μm/pixel, 488/525 nm [green], and 561/600 nm [red]). Mean intensity values for red and green fluorescence channels were obtained as part of region of interest (ROI) statistics in Nikon software. In a follow-up experiment, biofilms were grown for 3 days and then nonadherent material was removed, biofilms were washed once with PBS, fresh biofilm medium was added, and biofilms were incubated for an additional 3 days. Nonadherent material was removed and immunostained with MAbs 2-8G and 6-8C as described above.

### Competitive inhibition of S. mutans binding to human salivary agglutinin.

Unstimulated saliva was collected from healthy volunteers according to University of Florida (UF) institutional review board (IRB) protocol number 21-2004. Salivary agglutinin (SAG) was prepared as described previously, quantified via micro bicinchoninic acid (BCA) protein assay kit (ThermoFisher) using bovine serum albumin as the standard, and stored at −20°C until use (58). Microtiter plate wells were coated with 100 μL of a 40-μg/mL solution of SAG in PBS for 18 h at 4°C. Monomeric C123 and C123 fibers were prepared as described above. The ability of each form of the protein to competitively inhibit the binding of S. mutans to immobilized SAG was performed as described previously (29), except that assay plates were coated with SAG instead of collagen and that strain NG8 and the corresponding isogenic *ΔspaP* mutant were used. An uncoated well, incubated with PBS only, was used as a negative control to assess the background binding of S. mutans NG8, and a coated well without added bacteria was used as a blank for crystal violet *A*_595_ measurements. The amounts of added competitive inhibitor, C123 monomer, or amyloid fiber, ranged from 0.02 μg to 20 μg.

### Statistical analyses.

One-way analysis of variance (ANOVA) with Tukey’s *post hoc* comparison test was performed for ThT fluorescence and competitive binding assays (ns, not significant; *, *P* < 0.05; **, *P* < 0.01; ***, *P* < 0.001; ****, *P* < 0.0001). Confocal data were analyzed by two-way ANOVA followed by Bonferroni multiple-comparison test (***, *P* = 0.0001) and by Student's *t* test (*, *P* < 0.05).
