# Supplementary material for: Amyloid Aggregates Are Localized to the Nonadherent Detached Fraction of Aging Streptococcus mutans Biofilms
Source: Microbiol Spectr. 2022 Aug 11;10(4):e01661-22. doi: 10.1128/spectrum.01661-22 (PMC9431626; doi:10.1128/spectrum.01661-22)
Supplement: Supplemental file 1 — Supplemental material. Download spectrum.01661-22-s0001.pdf, PDF file, 6.6 MB [file spectrum.01661-22-s0001.pdf]

Supplemental Materials

Amyloid aggregates are localized to the non-adherent detached fraction of aging *Streptococcus mutans* biofilms

Running title: *S. mutans* amyloid location in biofilms

Elena Yarmola[1], Ivan P. Ishkov[1], Nicholas M. di Cologna[1], Megan Menashe[1], Robert L. Whitener[1], Joanna R. Long[3], Jacqueline Abranches[1], Stephen J. Hagen[2], L. Jeannine Brady[1#]

[1]Department of Oral Biology, University of Florida, Gainesville, Florida, 32610, USA.

[2]Department of Physics, University of Florida, Gainesville, Florida, 32610, USA.

[3]Department of Biochemistry and Molecular Biology, University of Florida, Gainesville, Florida, 32610, USA.

[#]Corresponding author: L. Jeannine Brady, Department of Oral Biology, University of Florida, P.O. Box 100424, Gainesville, Florida 32610

Email: jbrady@dental.ufl.edu

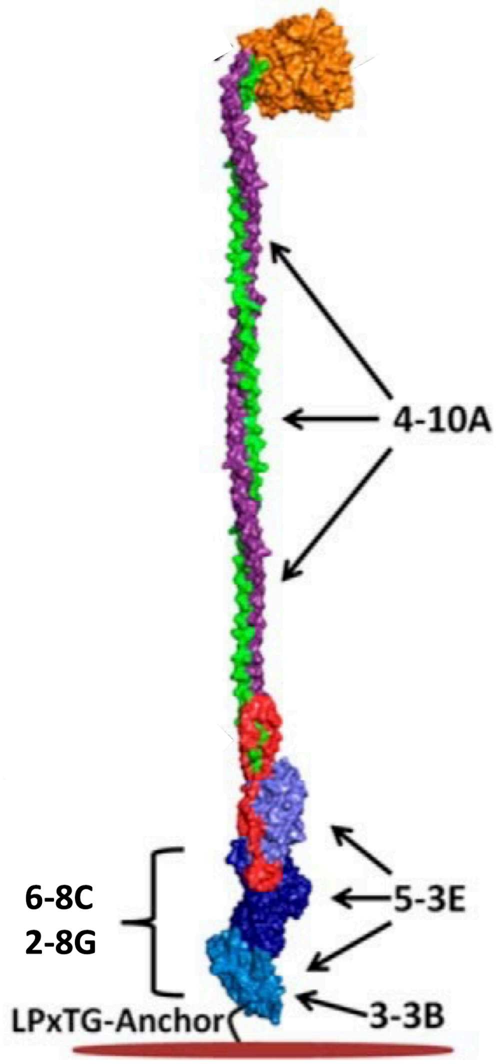

Figure S1. Approximate location of epitopes recognized by anti-P1 monoclonal antibodies used in this study.

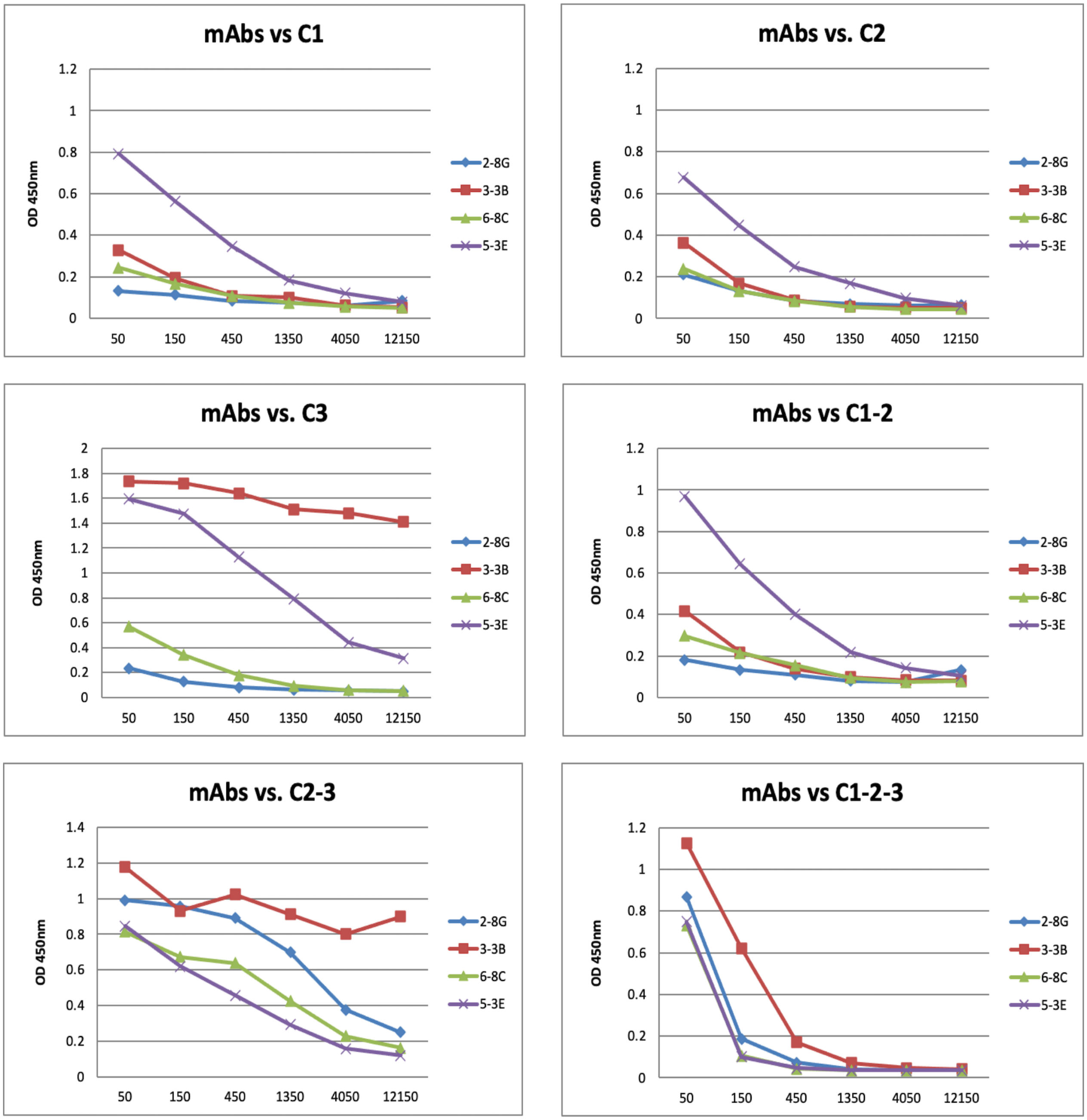

Figure S2. Evaluation of binding of anti-P1 monoclonal antibodies to C-terminal polypeptides by ELISA. The indicated dilution of mAb 6-8C, 2-8G, 5-3E, or 3-3B was reacted with 0.2 mg of recombinant C1, C2, C3, C12, C23, or C123 polypeptide immobilized in wells of 96-well Corning Costar flat bottom ELISA plates, followed by HRP-labelled goat anti-mouse secondary antibody

(Cappel) (1:1000) and development with *o*-phenylenediamine dihydrochloride substrate solution and measurement of absorbance at 450 nm. mAb 3-3B binds preferentially to the isolated C3 domain as well as the C23 polypeptide, while mAbs 6-8C and 2-8G bind better to the C23 polypeptide compared to the isolated C1, C2, or C3 domains, or to the C12 construct. mAb 5-3E reacted with every construct tested and appears to bind a repeated epitope present within C1, C2, and C3.

A

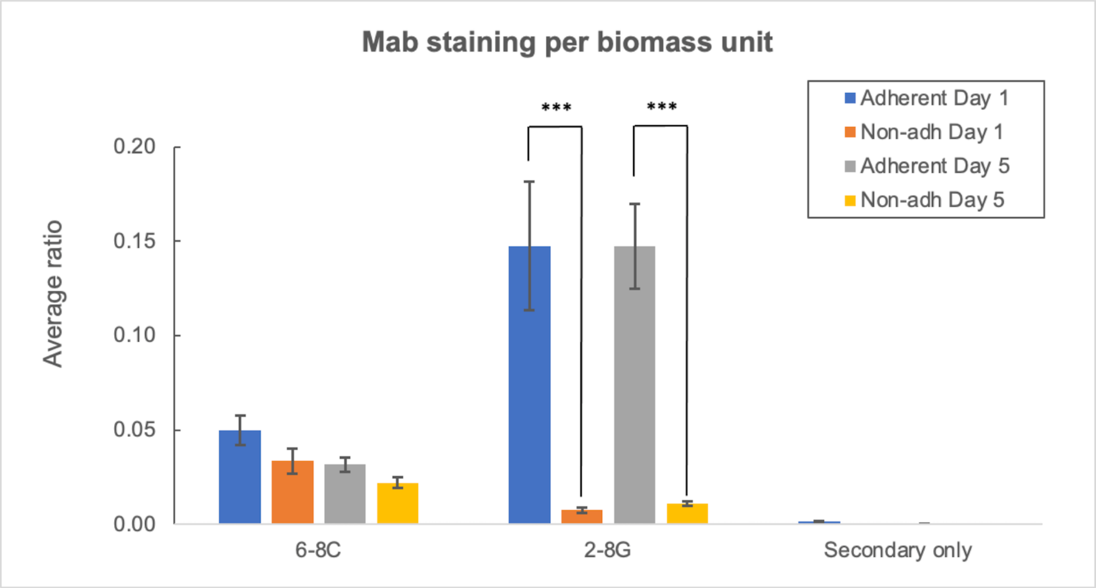

B

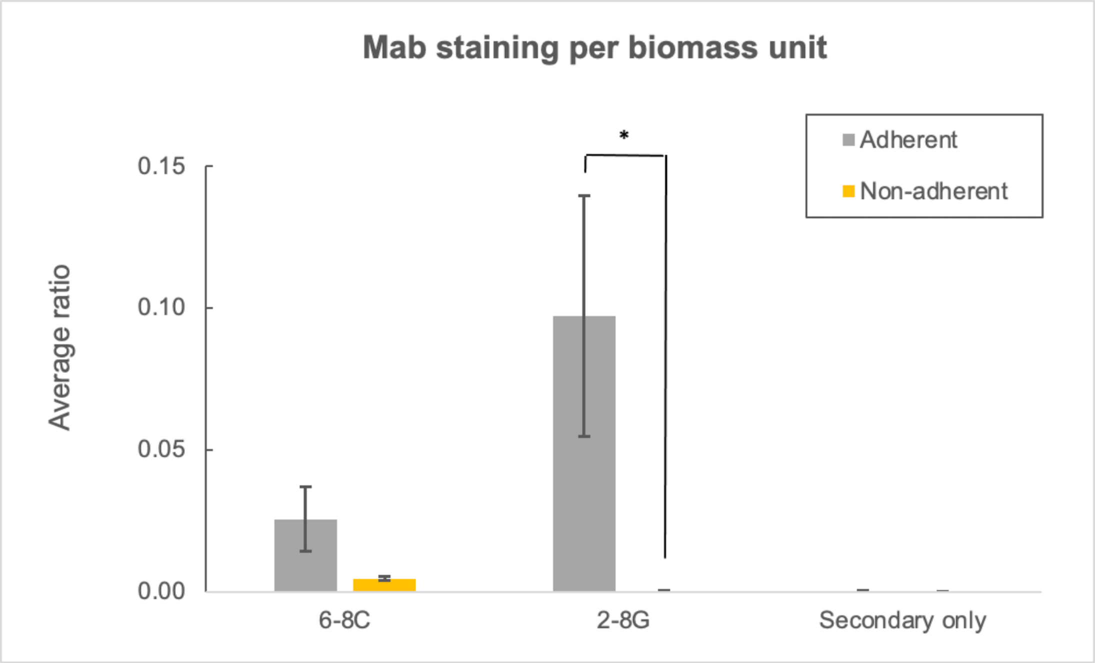

Figure S3. Quantification of confocal microscopy immunostained images of adherent and non-adherent fractions of *S. mutans* biofilms. A) Average ratios (+SEM) of MAb 6-8C and 2-8G immunostaining (red) compared to total biomass (GFP green fluorescence) for maximum

intensity projection images of adherent and non-adherent material in Day 1 and Day 5 samples (n>3). Data were analyzed by two-way ANOVA followed by Bonferroni multiple comparisons test (***p=0.0001). **B)** Average ratios (+SEM) of MAb 6-8C and 2-8G immunostaining (red) compared to total biomass (GFP green fluorescence) for maximum intensity projection images of non-adherent material from 6-day old biofilm samples in which all cellular and non-cellular material present in spent culture supernatants had been removed and replaced with fresh media on day 3 ($n \geq 6$). Data were analyzed by Student's t-test; *p=0.0206.

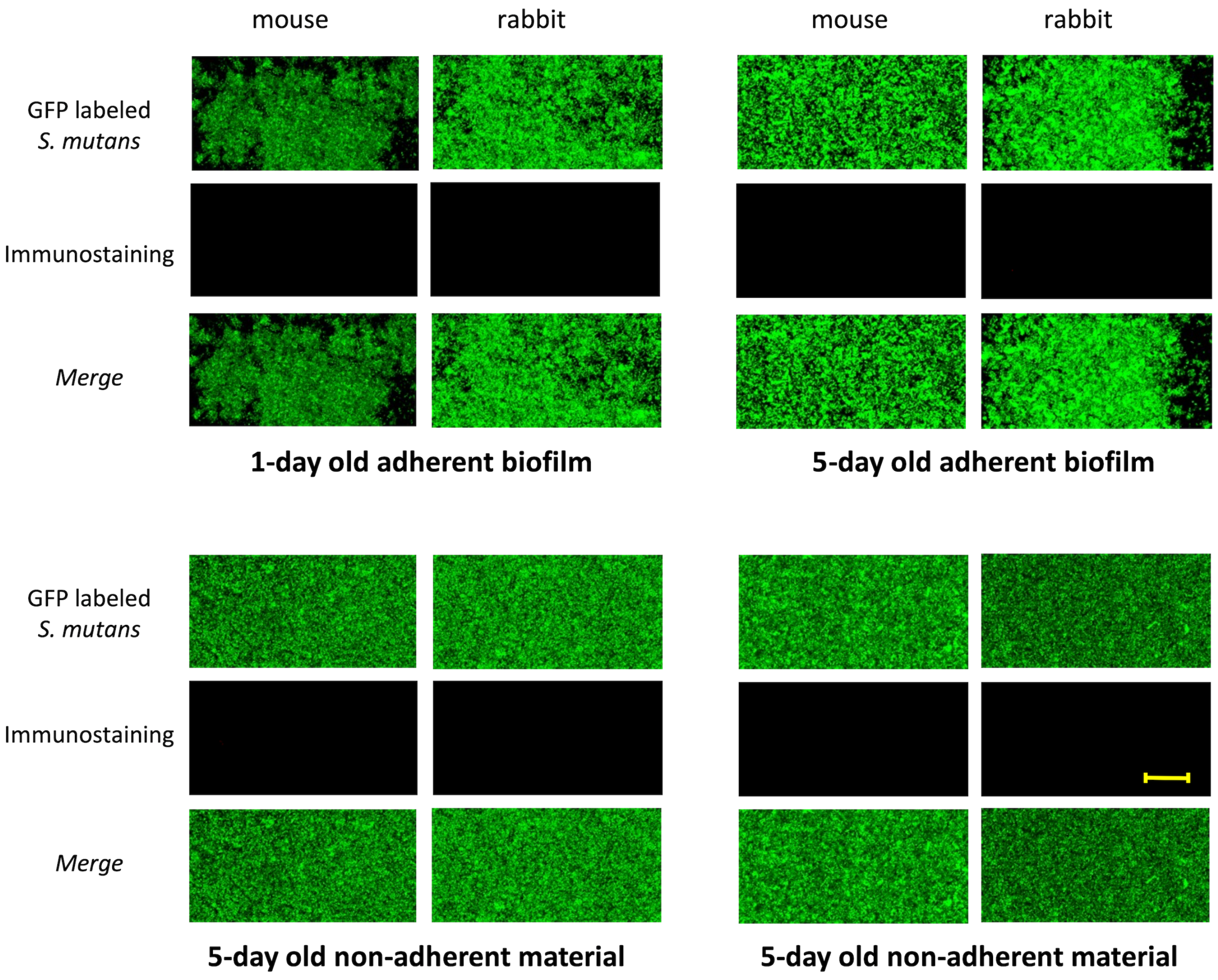

Figure S4. Secondary antibody only controls for confocal microscopy and immunostaining of adherent and non-adherent fractions of *S. mutans* biofilms. *S. mutans* (UA159::*Pldh-gfp*) biofilms were grown for one or five days then reacted with the indicated goat anti-mouse or anti-rabbit secondary antibody conjugated to Alexa Fluor® 594. Adherent fractions were stained directly on the slide on which the biofilm was grown. Non-adherent fractions were transferred to a tube and stained separately. Maximum intensity projections are shown. Scale bar: 20 μm.
